# Research on Temperature Control Method of Rice Noodles Extruder Based on APSO-MPC

**DOI:** 10.3390/s25123698

**Published:** 2025-06-13

**Authors:** Mengyao Zhang, Yunren Yang, Guohua Gao, Zhenlong Li, Yakai He, Peigang Li, Huangzhen Lv

**Affiliations:** 1 Chinese Academy of Agricultural Mechanization Sciences Group Co., Ltd., Beijing 100083, China; zmytumy@163.com (M.Z.); caams20220113@hotmail.com (Y.Y.); caamslzl@hotmail.com (Z.L.); 2 College of Mechanical and Energy Engineering, Beijing University of Technology, Beijing 100124, China; ggh6768@126.com; 3 China National Packaging and Food Machinery Co., Ltd., Beijing 100083, China18810809200@163.com (P.L.)

**Keywords:** rice noodle extrusion, temperature control, model predictive control, adaptive particle swarm optimization algorithm

## Abstract

Aiming to address the problems of many temperature control disturbances and the hysteresis of control output in existing rice noodle extruders, a temperature control method for a rice noodle extruder based on adaptive particle swarm optimization (APSO) optimization model predictive control (MPC) was designed. Firstly, the temperature control principle of the rice noodle extruder is analyzed by combining the structure of the rice noodle extruder. The temperature balance equation of the barrel is constructed by thermodynamic analysis, and the temperature prediction model is established. The APSO algorithm is further selected to perform the adaptive parameter identification of the model based on the collected input/output data. Then, aiming at high-precision temperature control, the objective function is constructed by combining the temperature prediction value and the reference trajectory, and the objective function is optimized to obtain the optimal control sequence. At the same time, the feed rate is selected as feedforward, the feed rate change is monitored by detecting the feed screw speed, and the optimal control sequence is corrected to eliminate the interference caused by the fluctuation of the feed rate. The experimental results show that the maximum temperature overshoot under different parameter combinations is 7.75%, the steady-state error is within ±1 °C, and the longest adjustment time is 1228 s. Compared with fuzzy PID control, it has stronger adaptability and higher control accuracy.

## 1. Introduction

With the rapid development of economic transformation and technology, the consumption of rice noodles in China is increasing year by year [1]. Rice noodle extruders are the core equipment for the production of rice noodles. Their main function is to make the raw rice noodle materials gelatinize and extrude under the action of shear extrusion and the external heating of the screw [2]. Since temperature has a significant effect on the gelatinization rate and gelatinization degree of rice noodles [3], the effect of the temperature control of the rice noodle extruder directly affects the quality of the finished rice noodles. The temperature of a rice noodle extruder is affected by many interference factors such as the screw speed, material characteristics, and feeding speed. The temperature of a rice noodle extruder fluctuates continuously in the actual production process.

For the temperature control of screw extruders, some of them use a PID controller to control the barrel temperature within the set range, but the effect is not good in the face of a large number of disturbing factors [4]. The most commonly used controllers are auto-tuned PID controllers, but traditional auto-tuning methods are mostly based on linear assumptions. For systems with significant nonlinearity and time lag, a combination of advanced control strategies is required. Therefore, the fuzzy PID control [5], generalized predictive control PID [6], fuzzy genetic algorithm [7], and other extruder temperature control methods have been developed. Schwarzinger present a hierarchical melt temperature controller for extruders. A rule-based layer adjusts cylinder zone temperatures based on the melt temperature error and a smart sensor’s estimated axial heat flow profile. This shapes the heat flow to achieve the desired melt temperature [8]. However, the above research focuses on the temperature control of a single barrel, which is not effective in the temperature control of the actual extrudate.

In order to improve the response speed, reduce the range of temperature fluctuation, and realize the precise control of the extruder temperature, Previdi proposed a prototype feedback controller, which realizes real-time temperature adjustment through two independent external single-input–single-output (SISO) control loops and seven identical internal SISO control loops [9]. Jiang proposed a temperature control for extruders based on multiple-input–multiple-output generalized predictive control (MIMO GPC). The barrel temperature model was obtained through theoretical analysis [10]. The feedforward controller and multivariable generalized predictive control were integrated, and the control performance was better than that of the PID controller. Therefore, the extruder as a multi-input–multi-output system can be more efficient and accurate in temperature control.

MPC is a mature control strategy. It is based on the mathematical model of the system, combined with predictive control, an optimization method, and feedback control. It realizes the control of the system by predicting the future behavior of the system and optimizing the control input. It can effectively deal with time-varying and multivariable complex systems and has many applications in precise temperature control [11,12,13,14]. Schwarzinger proposed an extruder temperature control based on MPC to determine the optimal temperature trajectory so as to achieve the best transition from the heating stage to the stable stage and realize the rapid conversion of the working point [15]. In summary, the construction of a multi-input and multi-output prediction model can effectively eliminate the lag of the extruder temperature control [16]. However, previous studies have not solved the problem of heat change and barrel coupling caused by the change of screw speed in the actual production process.

The purpose of this study is to realize the precise control of the temperature of a rice noodle extruder. Firstly, the MIMO temperature prediction model of the rice noodle extruder is constructed by the temperature balance equation. The APSO algorithm is used to identify the parameters of the model according to the collected input and output data. The optimal control sequence of the heater and the cooling sleeve is solved with a high-precision temperature control as the objective function. The feedforward control is used to eliminate the influence of the feed rate on the temperature, and an APSO-MPC rice noodle extruder temperature control method based on feedforward control is proposed. The accuracy of the temperature prediction model and the effectiveness of the temperature control method are verified by experiments.

## 2. Materials and Methods

### 2.1. Structure Composition of Machine

**Figure 1:**
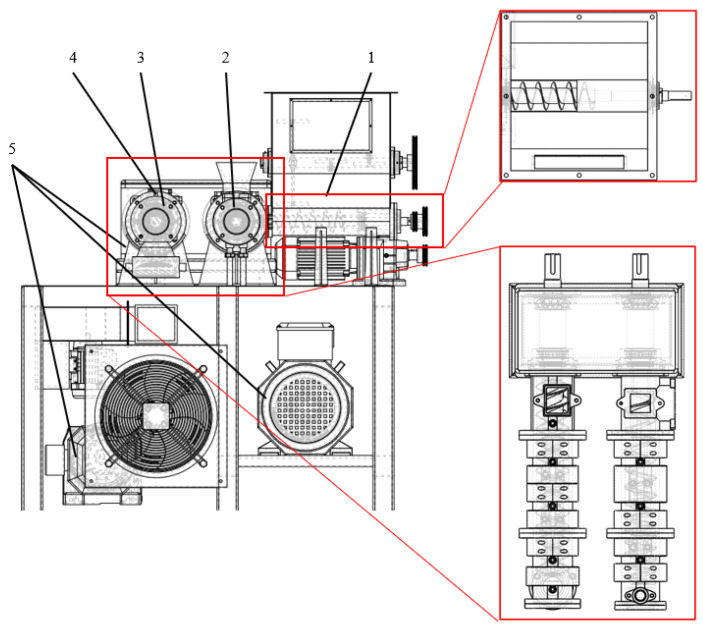
The main structure of the extruder, 1. Feeding device. 2. Ripening module. 3. Extrusion module. 4. Transmission module. 5. Heating and cooling module.

The main structure of the rice noodle extruder is shown in Figure 1. The main parameters of the extruder are shown in Table 1. It mainly includes a feeding device, a curing module, an extrusion module, a transmission module, and a heating and cooling device. The feeding device is mainly composed of a mixing barrel, a mixing shaft, and a feeding screw. The crushed rice noodles are evenly mixed in the mixing barrel and transported to the ripening module by the feeding screw. The ripening module and the extrusion module mainly complete the transportation and pressure building of the rice noodles through the screw, barrel, and die mouth so that the rice noodles are extruded from the die mouth according to the processing requirements. The transmission module is composed of a motor, a belt, and a spindle box, and the driving screw rotates at a specified speed. The heating and cooling module is the core of the temperature control system, which mainly includes a heater, a cooling sleeve, and a temperature sensor.

The temperature control system of the rice noodle extruder is divided into upper-computer and lower-computer PLC two-level control. The upper computer mainly completes the functions of parameter setting, data display, and data storage. The lower computer mainly completes the functions of data acquisition and program control. The SIMATIC S7-200 SMART PLC is selected as the lower computer, the expansion module is used to realize the input and output of the analog quantity, and Ethernet is used to connect with the upper computer. The system hardware mainly includes relays, resistance heaters, solenoid valves, cooling sleeves, temperature sensors, frequency converters, and drive motors. The screws of the feeding section, the curing section, and the extrusion section are controlled separately. The motor model is selected according to the requirements of each stage, and the appropriate frequency converter is selected to achieve accurate speed control. The temperature sensor selected is Heneng PT131 B, the temperature resolution is 0.1 °C, and the measured temperature value is read using the EM AT04 module. The heater adopts a resistance heater, the heating power is adjusted by a relay, the cooling sleeve is cooled by cooling water, and the cooling intensity is controlled by changing the opening of the flow valve.

**Figure 2:**
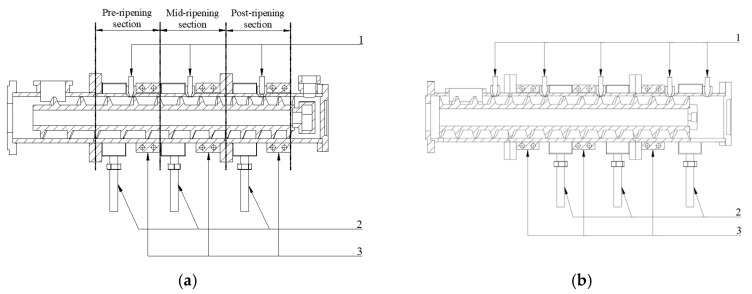
Arrangement of sensors and heating and cooling devices, 1. Temperature and pressure sensor. 2. Cooling device. 3. Heating device, (**a**) Ripening section, (**b**) Extruding section.

Since the feeding section does not need to control the temperature, the temperature sensor is evenly arranged on the barrel of the curing section and the extrusion section to detect the material temperature, and the heater and the cooling sleeve are arranged as the actuators of the temperature control. The arrangement of the sensor and the heating and cooling device is shown in Figure 2.

**Figure 3:**
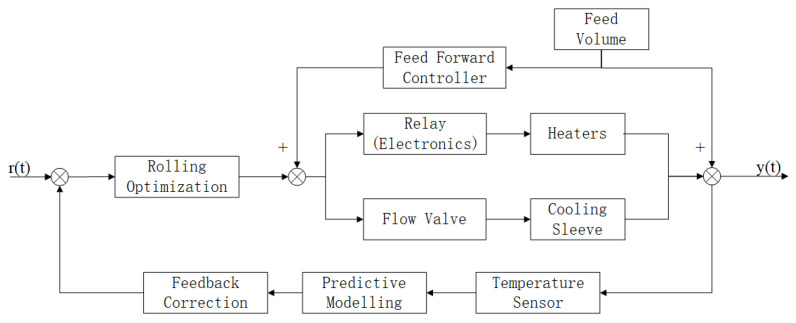
Temperature control structure of rice noodle extruder.

The temperature control principle of the rice powder extruder is shown in Figure 3. The barrel of the rice noodle extruder is regarded as a multi-input and multi-output system. The prediction model is constructed according to the temperature balance equation. The rolling optimization is carried out with high-precision temperature control as the objective function, and the optimal control amount is solved to control the heating and cooling device. The barrel temperature is mainly affected by the screw speed and feed rate. The screw speed is linearly related to the heat generated, and the screw speed is controlled as the input of the prediction model. The feed rate will affect the material filling degree in the barrel and the output of the rice noodle extruder. The feedforward control is used to eliminate the influence of the feed rate.

### 2.2. Temperature Prediction Model of Rice Noodle Extruder

Building a predictive model is required for achieving model predictive control. The predictive model needs to accurately capture the dynamic characteristics of the system and predict the future output behavior of the system based on the known data information in the past and the set future input [17]. Prediction models commonly employed in research encompass white-box models derived from physical laws, grey-box models integrating physical modeling with data-driven approaches, and black-box models reliant exclusively on data [18]. The grey box model balances the model accuracy and modeling cost [19] and has strong generalization. Therefore, this paper constructs the temperature balance equation of the barrel through the thermodynamic analysis of the barrel of the rice noodle extruder. On this basis, the temperature prediction model of the barrel is established, and the parameters of the model are identified according to the collected input and output data.

#### 2.2.1. Temperature Equilibrium Equation

The extrusion process of the rice powder extruder is essentially a comprehensive process of heat transfer, mass transfer, and physical coupling [20]. According to the law of conservation of energy, the temperature change in the barrel is equal to the heat inflow per unit time minus the heat outflow. Therefore, the heat change can be expressed as follows:

(1) $$\Delta Q=mc\frac{dT_{i}}{dt}$$

where

- ΔQ—heat change per unit time, W;
- *m*—mass of rice noodles in the barrel, kg;
- *c*—the specific heat capacity of rice noodles, J/(kg·K);
- $T_{i}$—*i* moment barrel temperature value, K.

Ignoring the amount of environmental interference, combined with the law of thermodynamics, the following can be obtained:

(2) $$\Delta Q=Q_{h}+Q_{s}-Q_{c}+Q_{l}+Q_{n}$$

where

- $Q_{h}$—the heat provided by the heater, W;
- $Q_{s}$—the heat generated by the screw rotation, W;
- $Q_{c}$—the heat taken away by the cooling sleeve, W;
- $Q_{l}$—the heat transferred from the previous cavity, W;
- $Q_{n}$—the heat transferred from the latter cavity, W.

The heat $Q_{h}$ provided by the heater is affected by the heater’s power $P_{h}$ and the heat transfer coefficient $K_{h}$, where the heater power is controlled by the input $u_{h}$. Therefore,

(3) $$Q_{h}=K_{h}P_{h}=K_{h}r_{h}u_{h}=K_{1}u_{h}$$

where

- $K_{h}$—the heat transfer coefficient;
- $P_{h}$—the heater power, W;
- $r_{h}$—the heating coefficient;
- $u_{h}$—the heater control volume.

The friction heat caused by screw rotation mainly comes from the relative motion between the material and the screw and the cylinder wall, $Q_{f}=\mu Fv$; due to the viscous dissipation generated by the internal viscous force during the flow process, the heat generated by the viscous dissipation can significantly affect the temperature control of the extrusion process, and sometimes it can even be used as the main heating source. The screw speed has a significant effect on the heat generated by the viscous dissipation. The two items are combined to make the output $u_{s}$ control the screw speed. Then, there IS

(4) $$Q_{s}=K_{s}r_{s}u_{s}=K_{2}u_{s}$$

where

- $K_{s}$—the viscous dissipation coefficient;
- $r_{s}$—the screw rotation coefficient;
- $u_{s}$—motor frequency, Hz.

The heat $Q_{c}$ taken away by the cooling sleeve is affected by the heat transfer coefficient $K_{c}$, the flow rate of the cooling water $dV_{c}/dt$, the specific heat $c_{c}$ of the cooling water, and other factors. The flow rate of the cooling water is affected by the degree of opening and closing of the valve; then, $dV_{c}/dt=r_{c}u_{c}$, and the rest are constants:

(5) $$Q_{c}=K_{c}c_{c}\frac{dV_{c}}{dt}\Delta T_{c}=K_{3}u_{c}$$

where

- $K_{c}$—the heat transfer coefficient of cooling sleeve;
- $c_{c}$—specific heat capacity of cooling water, J/(kg·K);
- $\frac{dV_{c}}{dt}$—flow rate of cooling water, m^3^/s;
- $\Delta T_{c}$—temperature difference between cooling water and material, K;
- $u_{c}$—valve control volume.

The heat $Q_{l}$ and $Q_{n}$ from the front and rear cavities are proportional to the cavity temperature; then, there are

(6) $$Q_{l}=K_{l}T_{i-1}$$

(7) $$Q_{n}=K_{n}T_{i+1}$$

where

- $K_{l},\;K_{n}$—heat transfer coefficient of front and rear cavity;
- $T_{i-1},\;T_{i+1}$—The temperature of the front and rear cavities, °C.

By sorting out Equations (1)–(7), we can get

(8) $$mc\frac{dT_{i}}{dt}=K_{1}u_{h}\left(t\right)+K_{2}u_{s}\left(t\right)-K_{3}u_{c}\left(t\right)+K_{l}T_{i-1}+K_{n}T_{i+1}$$

#### 2.2.2. Model Establishment

The input command of heating/cooling and the screw speed are selected as the input $u_{i}$ and the barrel temperature is selected as the output $y_{i}$ to construct a multi-input and multi-output model. The barrel temperature is measured by a K-type thermocouple, and the screw speed is calculated by the motor frequency. According to the temperature equilibrium equation,

(9) $$\dot{y}\left(t\right)=Ay\left(t\right)+Bu\left(t\right)$$

where

- $\dot{y}\left(t\right)$—output variation;
- $y\left(t\right)$—output matrix;
- $u\left(t\right)$—input matrix;
- A—output change matrix;
- B—input change matrix.

In order to distinguish the control variable and the disturbance variable in the input variable u, they are expressed. Equation (9) can be rewritten as

(10) $$\dot{y}\left(t\right)=Ay\left(t\right)+B_{1}u\left(t\right)+B_{2}w\left(t\right)$$

The state space equations are discretized by Euler’s method

(11) $$\dot{y}\left(t\right)=\frac{y\left(k+1\right)-y\left(k\right)}{T}$$

The state space model of the discrete-time system can be obtained by discretizing the above equation:

(12) $$y\left(k+1\right)=\bar{A}y\left(k\right)+{\bar{B}}_{1}u\left(k\right)+{\bar{B}}_{2}w\left(k\right)$$

where

- $\bar{A}=TA+I=\left[\begin{array}{cccccc} 1 & T\alpha_{12} & 0 & 0 & 0 & 0\\ T\alpha_{21} & 1 & T\alpha_{23} & 0 & 0 & 0\\ 0 & T\alpha_{32} & 1 & 0 & 0 & 0\\ 0 & 0 & 0 & 1 & T\alpha_{45} & 0\\ 0 & 0 & 0 & T\alpha_{54} & 1 & T\alpha_{56}\\ 0 & 0 & 0 & 0 & T\alpha_{65} & 1 \end{array}\right]$;
- ${\bar{B}}_{1}=T{\bar{B}}_{1}=\left[\begin{array}{cccccc} T\beta_{1} & 0 & 0 & 0 & 0 & 0\\ 0 & T\beta_{2} & 0 & 0 & 0 & 0\\ 0 & 0 & T\beta_{3} & 0 & 0 & 0\\ 0 & 0 & 0 & T\beta_{4} & 0 & 0\\ 0 & 0 & 0 & 0 & T\beta_{5} & 0\\ 0 & 0 & 0 & 0 & 0 & T\beta_{6} \end{array}\right]$;
- ${\bar{B}}_{1}=T{\bar{B}}_{1}=\left[\begin{array}{cc} T\beta_{s11} & 0\\ T\beta_{s12} & 0\\ T\beta_{s13} & 0\\ 0 & T\beta_{s21}\\ 0 & T\beta_{s22}\\ 0 & T\beta_{s23} \end{array}\right]$.

#### 2.2.3. APSO Identification Model Parameters

**Figure 4:**
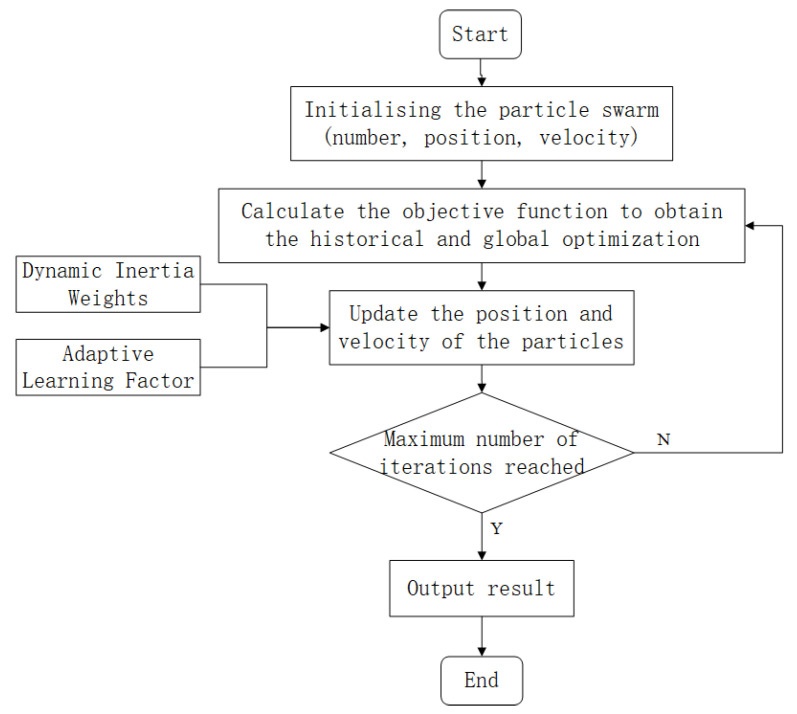
Flowchart of the APSO algorithm.

In recent years, in addition to conventional mathematical optimization methods such as the least squares method, many heuristic algorithms have been widely used in the field of model parameter identification [21,22], such as the genetic algorithm, the particle swarm optimization algorithm, and the simulated annealing algorithm. Among them, the particle swarm optimization algorithm is simple in principle, fast in convergence, and relatively small in parameter adjustment and has strong adaptability in complex search space [23]. Therefore, the particle swarm optimization algorithm is selected to optimize the parameters of the model. Since the particle swarm optimization algorithm easily falls into local optimal solutions, the dynamically adjusted inertia weight is introduced; that is, the inertia weight gradually decreases with the increase of the number of iterations. The global search is carried out in the early stage, the optimal solution is gradually converged in the later stage, and the learning factor is adaptively adjusted to balance the global search and local search capabilities. The APSO algorithm flow is shown in Figure 4.

The extrusion test was carried out with an MFJ110 rice noodle extruder as the test equipment. The heater output, cooling water flow, screw speed, and material temperature were taken as data acquisition objects, and the sampling period was 1 s. The temperature data is read by the Siemens EM AT04 module, which has the ability to suppress noise and adopts the integral measurement method to average the signal in the specified time period and improve the anti-interference ability. The suppression frequency is 50 Hz, the integration time is 20 ms, and the module update time is 0.263 s. The screw speed is obtained by reading the frequency of the frequency converter. When the motor speed is too low, the shear force of the screw on the material in the cavity decreases, resulting in incomplete ripening of rice noodles. When the motor speed is too high, the rice noodles in the cavity is excessively gelatinized, resulting in rice noodles burnt [24]. Therefore, the frequency range of the curing screw and extrusion screw motor is 20~35 Hz. The heater output, cooling water flow, and screw speed are modified for data acquisition.

Firstly, the dimension and upper and lower bounds are determined according to the unknown parameters of the model and their value ranges, and the population is randomly initialized to determine the population size and evolutionary termination conditions. The objective function is constructed according to the temperature output value predicted by the model and the mean square error MSE of the actual data:

(13) $$f=\frac{1}{N}\overset{N}{\underset{k=1}{\sum }}{\left\|y_{k}-{\hat{y}}_{k}\right\|}_{2}$$

where

- *N*—number of input samples;
- $y_{k}$—the actual temperature at the kth sampling time;
- ${\hat{y}}_{k}$—predicted temperature at the kth sampling time.

**Figure 5:**
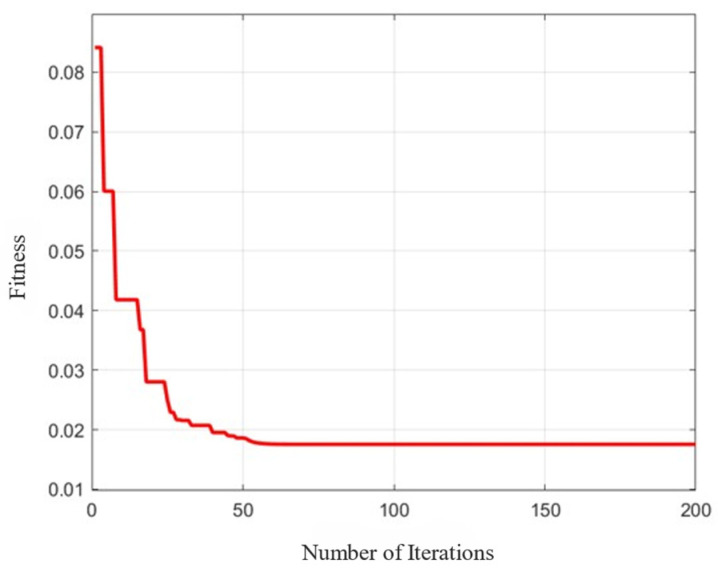
APSO iteration profile.

The APSO algorithm sets the population size to 50, the maximum and minimum inertia parameters to 0.9 and 0.4, and the maximum number of iterations to 200. As shown in Figure 5, as the number of iterations increases, the fitness function value decreases rapidly, and in the first 50 iterations, the fitness function value decreases most significantly. After 200 iterations, the fitness function value stabilizes, indicating that it is close to the optimal parameter identification result at this time.

In order to verify the accuracy of the prediction model, 3600 sets of data with a sampling time of 1 s were selected as the input of the model, and the barrel temperature obtained by the prediction model was compared with the measured temperature. In order to quantitatively analyze the prediction accuracy and fitting degree of the model, three evaluation indexes are selected to evaluate the performance of the model, namely RMSE, MAE, and $R^{2}$. The calculation results are that RMSE is 0.2998, MAE is 0.5589, and $R^{2}$ is 0.928, indicating that the model has high prediction accuracy and can reflect the dynamic change law of rice noodle extruder temperature.

### 2.3. Design of Model Predictive Control

#### 2.3.1. Rolling Optimization

The rolling optimization means that after designing the cost function according to the actual demand, the optimal control sequence is solved online by minimizing the cost function, the first step in the control sequence is taken as the current control quantity, and the optimal control is repeatedly solved at the subsequent sampling time. Repeated online optimization reduces the influence of abnormal conditions such as varying time and the disturbance of the controlled object so that the control quantity of each step is optimal and the good performance of the system is maintained.

According to the temperature prediction model of the rice noodle extruder, the control quantity and output quantity in N sampling periods in the future can be deduced:

(14) $$Y=Gy\left(k\right)+H_{1}U+H_{2}W$$

where

- $Y={\left[\begin{array}{cccc} y\left(k+1\right) & y\left(k+2\right) & \ldots & y\left(k+N\right) \end{array}\right]}^{T}$;
- $U={\left[\begin{array}{cccc} u\left(k\right) & u\left(k+1\right) & \ldots & u\left(k+N-1\right) \end{array}\right]}^{T}$;
- $W={\left[\begin{array}{cccc} w\left(k\right) & w\left(k+1\right) & \ldots & w\left(k+N-1\right) \end{array}\right]}^{T}$;
- $G={\left[\begin{array}{cccc} \bar{A} & {\bar{A}}^{2} & \ldots & {\bar{A}}^{N} \end{array}\right]}^{T}$
- $H_{1}=\left[\begin{array}{ccccc} {\bar{B}}_{1} & 0 & 0 & \ldots & 0\\ \bar{A}{\bar{B}}_{1} & {\bar{B}}_{1} & 0 & \ldots & 0\\ {\bar{A}}^{2}{\bar{B}}_{1} & \bar{A}{\bar{B}}_{1} & {\bar{B}}_{1} & \ldots & 0\\ \vdots & \vdots & \vdots & \ddots & \vdots \\ {\bar{A}}^{N} & {\bar{A}}^{N-1}{\bar{B}}_{1} & {\bar{A}}^{N-2}{\bar{B}}_{1} & \ldots & {\bar{B}}_{1} \end{array}\right]$;
- $H_{2}=\left[\begin{array}{ccccc} {\bar{B}}_{2} & 0 & 0 & \ldots & 0\\ \bar{A}{\bar{B}}_{2} & {\bar{B}}_{2} & 0 & \ldots & 0\\ {\bar{A}}^{2}{\bar{B}}_{2} & \bar{A}{\bar{B}}_{2} & {\bar{B}}_{2} & \ldots & 0\\ \vdots & \vdots & \vdots & \ddots & \vdots \\ {\bar{A}}^{N} & {\bar{A}}^{N-1}{\bar{B}}_{2} & {\bar{A}}^{N-2}{\bar{B}}_{2} & \ldots & {\bar{B}}_{2} \end{array}\right]$.

In order to make the expected value of the model output reach the set temperature $y_{set}$ smoothly, the softening coefficient α is selected to construct the reference trajectory. The optimization goal of the system is to reduce the error between the predicted output and the reference trajectory and minimize the energy consumption of the system. According to the optimization goal, the objective function is constructed by combining the temperature prediction value and the reference trajectory as follows:

(15) $$J=\overset{n}{\underset{j=1}{\sum }}Q{\left[y\left(k+j\right)-y_{c}\left(k+j\right)\right]}^{2}+\overset{m}{\underset{j=1}{\sum }}R{\left[u\left(k+j\right)\right]}^{2}$$

where

- Q—Weight matrix of temperature error;
- R—Weight matrix of energy consumption.

In order to prevent the control variables from exceeding the rated control value, constraints are added to the control variables according to the actual situation. The motor frequency control range is 20 ~ 35 Hz, and the heating and cooling control range is [−1, 1]. After integrating the objective function and constraints, the following optimization problems can be obtained:

(16) $$\left\{\begin{array}{c} minJ=U^{T}\left(H^{T}QH+R\right)U+2{\left(Gy\left(k\right)-Y_{c}\right)}^{T}QHU\\ U_{min}\leq U\leq U_{max} \end{array}\right.$$

#### 2.3.2. Feedback Correction

In order to improve the accuracy of model predictive control, considering external disturbances, model inaccuracy, and other factors, the model predictive output is corrected by the error between the predictive model output and the actual system output to compensate the predictive error [25]. At time k, the prediction error is

(17) $$e\left(k\right)=y\left(k\right)-{\hat{y}}_{r}\left(k\right)$$

where

- $y\left(k\right)$—the actual output at the current moment;
- ${\hat{y}}_{k}\left(k\right)$—the output of model prediction.

### 2.4. Feedforward Control

The feedforward control system can directly overcome the influence of the disturbance on the controlled quantity by detecting the change in the disturbance quantity [26]. The feed rate is selected as feedforward, and the feed rate change is monitored by detecting the rotation speed of the feed screw. The feedforward control function is calculated by the following formula.

(18) $$G_{b}\left(s\right)=-\frac{G_{f}\left(s\right)}{G_{o}\left(s\right)}$$

where

- $G_{f}\left(s\right)$—interference transfer function;
- $G_{o}\left(s\right)$—control transfer function.

The interference transfer function and the control transfer function are approximated as the first-order pure lag function. For the ripening section subjected to step-response testing, a 90 °C temperature setpoint was applied; the resulting transient response was analyzed via least-squares estimation to parameterize a first-order transfer function, yielding the model in Equation (19).

(19) $$G_{o}\left(s\right)=\frac{5.1}{337s+1}e^{-57s}$$

Similarly, the feed motor frequency is set to 25 Hz to obtain the temperature response curve under the step change of the feed rate, and the least squares method is used to fit the function parameters to obtain the model shown in Equation (20).

(20) $$G_{f}\left(s\right)=\frac{e^{-85s}}{34s+1}$$

The transfer function of the feedforward controller is

(21) $$G_{b}\left(s\right)=-0.2\frac{337s+1}{34s+1}e^{-28s}$$

## 3. Results

### 3.1. Simulation Results and Analysis

#### 3.1.1. Objective Function Weight Selection

Because the temperature control accuracy directly affects the quality of rice noodles, the control error of reducing the barrel temperature has a higher weight when controlling the temperature of the rice noodle extruder, and the temperature control priority of each barrel is the same. The weight coefficient of the energy consumption r = 0.01 is fixed, and the weight coefficient of the temperature error q is changed. The simulation experiment is carried out to compare the time when the barrel temperature reaches the target value (90 °C) under different weight coefficients.

It can be seen from Table 2 that as the *q* value ranges from 0.5 to 5, the time for the barrel temperature to reach the target value decreases with the increase in the *q* value. When the *q* value exceeds 10, the time when the barrel temperature reaches the target value tends to be stable, and there is no significant reduction. Therefore, the temperature error weight coefficient *q* = 10 is selected to reduce the energy consumption while improving the control accuracy.

#### 3.1.2. Dynamic Performance Simulation Analysis

**Figure 6:**
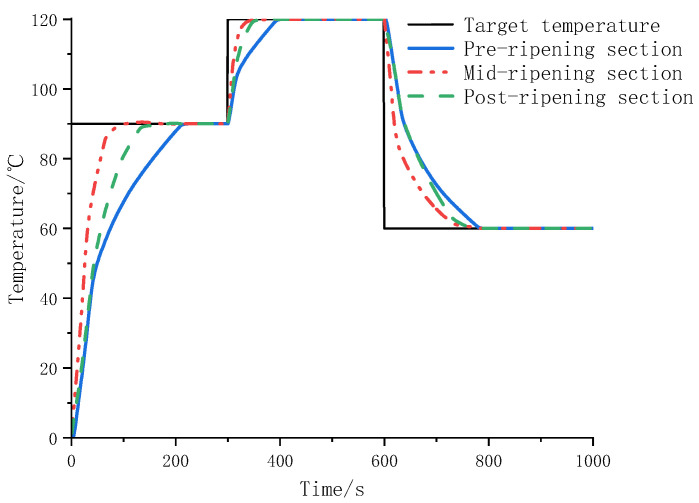
Temperature response curve under model predictive control.

The temperature control model of the rice noodle extruder was established in MATLAB R2022a. The parameters of the control model were set according to the above method. The prediction time domain *p* was 10, the control time domain m was 6, the temperature error weight coefficient *q* was 10, and the energy consumption weight coefficient r was 0.01. The frequency of the constrained motor was 20~35 Hz, the heating and cooling control range was −1~1, the temperature range was −50~200 °C, and the target temperature value changed with time. The simulation results are shown in Figure 6.

When the target value is set to 90 °C, the stabilization times are 213 s, 199 s, and 191 s; the steady-state error is 0; and the maximum overshoot is 0.19%. In the range of 0~300 s, the heating control quantities were 151.90, 71.13, and 171.26. After adjusting the target temperature to 120 °C, the system reached a stable state in 91 s, 90 s, and 88 s. Within 300–600 s, the heating control quantities were 121.51, 25.31, and 186.62. After adjusting the target value to 60 °C, the system reached a stable state within 172 s, 166 s, and 171 s. Within the 600~900 s, the cooling control amounts were 23.38, 18.64, and 66.34.

The results showed that the curing front section was connected with the feed screw, and the main function quickly increased the temperature of rice noodles, so the heating rate was slow, the heating control amount was large, and the cooling control amount was small. The middle stage of curing was affected by the temperature of the two stages, the heating speed was fast, and the heating control amount was small. The temperature fluctuation in the later stage of curing was the largest, and the heating control amount and cooling control amount were large to reduce the temperature fluctuation. In general, the system can smoothly transition to the temperature setting value without steady-state error and has good dynamic and steady-state performance.

### 3.2. Test Validation

#### 3.2.1. Analysis of Control Effects

In order to evaluate the performance of the designed temperature control method, the MFJ110 rice noodle extruder was used as the test equipment for the verification test. The temperature value in the PLC was through S7-200 PC Access SMART, Matlab was set as the OPC client, the OPC configuration module in the Simulink component library was selected, and groups and variables were added. The communication between Matlab and PLC was established, the temperature sensor value was read in PLC, and MPC calculation was performed. The result of the operation was returned to PLC, and the control of the heater and cooler was performed.

The target temperatures were set to 80, 90, and 100 °C, and the ripening section of the screw motor frequency was set to 20 Hz, 25 Hz, and 30 Hz, a total of nine groups of parameter combinations were set for the test. The temperature control overshooting, steady state error, and regulation time were calculated as shown in Table 3. The results show that the temperature overshooting amount controlled by the APSO-MPC control algorithm is up to 7.75%, the steady-state error is within ±1 °C, the regulation time is limited by the power of the heater, and the longest time is 1228 s. The results show that the designed method of controlling the temperature of the rice noodle extruder has the advantages of a small overshooting amount, a short regulation time, and no oscillating phenomenon, and it is able to realize fast and stable control.

#### 3.2.2. Comparative Tests

In order to further verify the superiority of the APSO-MPC temperature control algorithm, fuzzy PID control was used to carry out the tests of the above nine sets of parameter combinations, and the temperature control results were compared with the designed APSO-MPC control. The fuzzy PID sets the temperature error E to vary in the range of [0, 120] °C and the temperature error change rate Ec to vary in the range of [−0.1, 0.16] °C/s, using the triangular subordination function.

**Figure 7:**
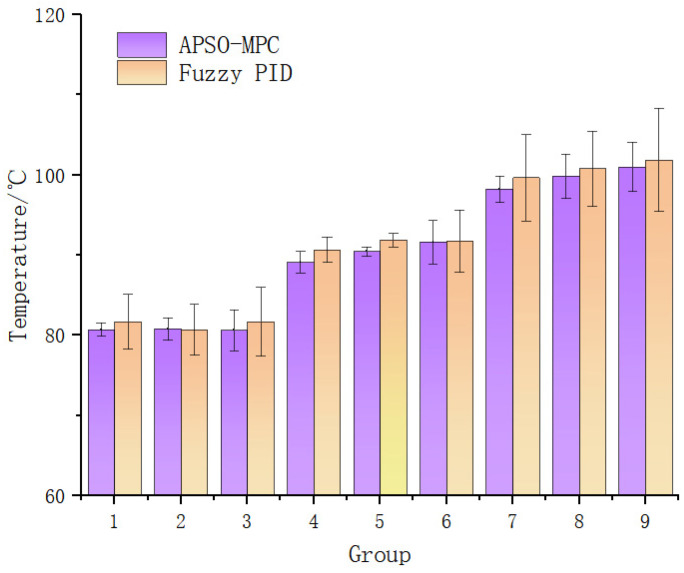
Control performance comparison.

The results are shown in Figure 7. The fuzzy PID control performed well when the target temperature value was 90 °C and the motor frequency was 25 Hz, and the error value was only 0.841 °C, which is not much different from the APSO-MPC control. However, as the target temperature and motor frequency changed, the external disturbances such as friction heat and viscous dissipation of rice noodles became larger. At this time the effect of fuzzy PID control was weakened, and the maximum error value reached 6.415 °C. Compared to fuzzy PID, the APSO-MPC controller demonstrates smaller error fluctuations (maximum 3.065 °C) and reduced variation across parameter configurations, suggesting improved robustness and higher control accuracy.

## 4. Discussion

In this study, the rice noodles extruder is regarded as a multi-input and multi-output system, and a prediction model is established by combining the thermodynamic analysis. The results show that, compared with the single-input and single-output model, the model constructed by this method treats the whole rice noodles extruder as a whole, takes into account the influences of multiple interfering factors, and excludes them so that the prediction of temperature is more efficient and accurate. However, the model has a strong specificity for different structures of the extruder to be modeled separately.

At the same time, compared with fuzzy PID control and other control methods commonly used in extruders, the model predictive control can better eliminate the control hysteresis and heating inertia due to the deviation of the heating and cooling device of the rice noodles extruder and the position of the temperature control point, as well as the long heat transfer time of the heater. However, this study did not use complex optimization algorithms considering the real-time nature of the control, and more advanced optimization algorithms can also be considered in subsequent studies to complete the rolling optimization part of the model predictive control.

## 5. Conclusions

In this study, for the temperature control system of a rice noodle extruder, a high-precision temperature prediction model is constructed based on thermodynamic analysis, the APSO algorithm is used to complete the parameter identification, and the APSO-MPC method incorporating feed-forward control is innovatively proposed as follows.

(1) The thermodynamic analysis of the barrel of rice noodle extruder was carried out, the temperature equilibrium equation was obtained, and the mechanism model of temperature prediction was established. The rice noodle extrusion test was carried out, and the data of the heater control, cooling sleeve control, screw speed, and barrel temperature were collected. The APSO algorithm was used to identify the parameters of the temperature prediction model. A total of 3600 sets of input and output data were selected for the verification test of the model. The results showed that the RMSE was 0.2998, the MAE was 0.5589, and the $R^{2}$ was 0.9238, indicating that the model had high prediction accuracy.

(2) An APSO-MPC temperature control method based on feed-forward control is proposed, using feed-forward control to eliminate the influence of feed quantity, determining the weight coefficients of the objective function, and realizing the simulation control of the temperature of the barrel of the rice noodle extruder by using the heating and cooling of each section of the barrel as the controlling quantity and the screw rotational speed as the measurable interference. The simulation results show that when the target temperature is 90 °C, the fastest adjustment time of the designed temperature control method is 191 s, the steady-state error is 0, and the maximum overshooting amount is 0.19%, which indicates that the designed temperature control method has a good dynamic steady-state performance.

(3) By changing the target temperature value and motor frequency and other parameters for experimental verification, and with the fuzzy PID control for a comparison test, the results show that the temperature overshooting under different parameter combinations is up to 7.75%, the steady-state error is within ±1 °C, and the regulation time is up to 1228 s, compared with the fuzzy PID control, for better adaptability and higher control accuracy.

## Figures and Tables

**Table 1:** Rice noodle extruder main parameter.

Item	Parameter Value
Specification	MFJ110
Diameter of sleeve	110 mm
L/D	12
Matching motor	GDVP180L-6 GDVP132M-4
Reduction rate	2.7

**Table 2:** Stabilization time of the system under the influence of different weights.

Weight of Temperature Error (*q*)	The Time When the System Reaches Stability (s)
0.5	257
1	231
2	217
5	206
10	199
50	198

**Table 3:** Performance parameters for different combinations of parameters.

Target Temperature (°C)	Motor Frequency (Hz)	Maximum Overshoot (%)	Steady State Error (°C)	Adjustment Time (s)
80	20	1.88	0.227	672
90	20	1.94	0.289	982
100	20	2.80	0.805	1228
80	25	2.96	0.641	644
90	25	5.14	0.638	756
100	25	4.75	0.548	957
80	30	4.38	0.736	612
90	30	6.89	0.811	733
100	30	7.25	0.778	903

## Data Availability

The raw data supporting the conclusions of this article will be made available by the authors upon request.

## Abbreviations

The following abbreviations are used in this manuscript:

APSO	Adaptive Particle Swarm Optimization
MPC	Model Predictive Control
SISO	Single-Input Single-Output
MIMO	Multiple Input Multiple Output
GPC	Generalized Predictive Control
RMSE	Root Mean Square Error
MAE	Mean Absolute Error
OPC	OLE for Process Control
